# [18F]Fludeoxyglucose-Positron Emission Tomography Evidence for Cerebral Hypermetabolism in the Awake State in Narcolepsy and Idiopathic Hypersomnia

**DOI:** 10.3389/fneur.2017.00350

**Published:** 2017-07-20

**Authors:** Yves Dauvilliers, Elisa Evangelista, Delphine de Verbizier, Lucie Barateau, Philippe Peigneux

**Affiliations:** ^1^Centre de Référence Nationale Maladie Rare, Narcolepsie et Hypersomnie Idiopathique, Unité de Sommeil, Hôpital Gui-de-Chauliac, CHU Montpellier, Montpellier, France; ^2^INSERM U1061, Montpellier, France; ^3^Service de Médecine Nucléaire, Hôpital Gui-de-Chauliac, CHU Montpellier, Montpellier, France; ^4^UR2NF, Neuropsychology and Functional Neuroimaging Research Unit, Centre de Recherches Cognition et Neurosciences (CRCN), ULB Neurosciences Institute (UNI), Université Libre de Bruxelles, Brussels, Belgium

**Keywords:** narcolepsy, idiopathic hypersomnia, metabolism, positron emission tomography, imaging, salience network, default mode network, executive-control network

## Abstract

**Background:**

Changes in structural and functional central nervous system have been reported in narcolepsy, with large discrepancies between studies. No study has investigated yet spontaneous brain activity at wake in idiopathic hypersomnia (IH). We compared relative changes in regional brain metabolism in two central hypersomnia conditions with different clinical features, namely narcolepsy type 1 (NT1) and IH, and in healthy controls.

**Methods:**

Sixteen patients [12 males, median age 30 years (17–78)] with NT1, nine patients [2 males, median age 27 years (20–60)] with IH and 19 healthy controls [16 males, median age 36 years (17–78)] were included. ^18^F-fludeoxyglucose positron emission tomography (PET) was performed in all drug-free subjects under similar conditions and instructions to stay in a wake resting state.

**Results:**

We found increased metabolism in the anterior and middle cingulate and the insula in the two pathological conditions as compared to healthy controls. The reverse contrast failed to evidence hypometabolism in patients vs. controls. Comparisons between patient groups were non-significant. At sub-statistical threshold, we found higher right superior occipital gyrus glucose metabolism in narcolepsy and higher middle orbital cortex and supplementary motor area metabolism in IH, findings that require further confirmation.

**Conclusion:**

There is significant hypermetabolism in narcolepsy and IH in the wake resting state in a set of brain regions constitutive of the salience cortical network that may reflect a compensatory neurocircuitry activity secondary to sleepiness. Metabolic differences between the two disorders within the executive-control network may be a signature of abnormally functioning neural system leading to persistent drowsiness typical of IH.

## Introduction

In the past few decades, neuroimaging studies have significantly contributed to our understanding of sleep physiology and sleep disorders in humans (1). Changes in structural and functional central nervous system have been reported in central hypersomnia disorders and especially in narcolepsy (2–6). However, results are inconsistent with either increased or decreased relative regional metabolism and perfusion in particular areas. For instance, a ^18^F-fludeoxyglucose (FDG) positron emission tomography (PET) study reported reduced metabolism in narcolepsy in a wide set of cortico-subcortical areas including the bilateral rectal and subcallosal gyri, the right superior frontal gyrus and inferior parietal lobule, the left supramarginal gyrus, the precuneus bilaterally, and the posterior hypothalamus and mediodorsal thalamic nuclei (3). Using a similar procedure, we found, on the opposite, increased metabolism in the anterior and mid-cingulate cortex, the cuneus, and the lingual gyrus in drug-free patients with narcolepsy–cataplexy scanned in a fully awake condition (2). A more recent PET study found hypometabolism in the right mid-frontal lobe and angular gyrus in young type 1 narcoleptic patients and hypermetabolism in the hippocampus, parahippocampus, amygdala, fusiform, left inferior parietal lobe, left superior temporal lobe, striatum, basal ganglia and thalamus, right hypothalamus, and pons (6). Discrepancies between studies might be partially explained by differences between populations [controls and patients’ phenotype, narcolepsy type 1 (NT1) vs. 2, etc.], drug-free vs. treatment conditions, age and disease duration, sample size, procedure and scanning methods, and statistical analyses. However, we may also hypothesize that most of these differences actually relate to a dissimilar vigilance status during the imaging sessions (i.e., awake vs. drowsy vs. sleepy).

Spontaneous ensembles of brain activity in the resting state are classically categorized between three major networks according to their underlying neuroanatomy and functions: (1) the default-mode network that relates to internally oriented thoughts and whose major node is the posterior cingulate cortex, (2) the salience network linking the detection of behaviorally relevant stimuli and the coordination of neural resources, mostly associated with the anterior cingulate and insular cortices nodes, and (3) the executive-control network that relates to externally guided awareness, linked with dorsal lateral prefrontal cortex and precuneus nodes (7–10). Considering in this framework, the results of the three aforementioned studies (2, 3, 6), patients with NT1 may exhibit decreased activity in part of the executive-control network and/or increased activity in part of the salience network. Importantly, relative hyper- or hypometabolism might be contingent upon the conditions and instructions given to the subjects at the time of PET scanning, more than to the development of the disorder in itself and/or the consequences of the sleep pathology.

Narcolepsy type 1 is the most severe cause of daytime sleepiness in humans, associated with cataplexy, frequent disrupted nighttime sleep, hypnagogic hallucinations and sleep paralysis, with recent insights demonstrating selective and irreversible loss of hypothalamic hypocretin neurons (11, 12). Idiopathic hypersomnia (IH) is another orphan central hypersomnolence disorder, with no identified biomarkers so far (13). Management strategies for hypersomnolence are rather codified for both disorders with first-line medication being modafinil, with potential for innovative emerging therapies in the next few years (14–16).

To the best of our knowledge, no study has yet investigated spontaneous brain activity in the resting awake state in IH. Since activation and deactivation of particular central neural pathways are reported across different vigilance states (17), we compared intrinsic neural activity in the wake state using ^18^FDG PET-scan under similar instructions and conditions in drug-free patients with NT1 or with IH, and in healthy controls. Considering that hypersomnolence is a shared symptom in narcolepsy and IH, leading to higher demands to maintain a wake state, we hypothesized that fluctuations of brain’s intrinsic activity in patients might reflect the involvement of the neurocircuitry underlying sleepiness in the two pathological conditions, rather than a specific feature of the underlying disorder itself (e.g., the hypocretin deficiency for NT1). In other terms, patients with either NT1 or IH may exhibit significant brain metabolism differences in comparison to healthy controls, but with limited differences between the two patient groups.

## Materials and Methods

### Participants

Sixteen drug-free patients [12 males and 4 females, median age 30 years (range 17–78)] with NT1, clear-cut cataplexy, positive HLA DQB1*06:02 typing were included. All patients complained of constant EDS (Score on Epworth Sleepiness Scale >10/24). All patients had ≥2 sleep onset REM periods (SOREMPs) and 15 had mean sleep latency <8 min on the multiple sleep latency test. All had low CSF hypocretin-1 level (<110 pg/ml). Seven drug-free patients with NT1 were already included in our previous PET scan study (2).

Nine drug-free patients [2 males and 7 females, median age 27 years (range 20–60)] affected with IH according to ICSD-3 criteria were included (18). All patients complained of constant excessive daytime sleepiness (Score on Epworth Sleepiness Scale >10/24), non-refreshing naps irrespective of duration, uninterrupted and prolonged nighttime sleep (>10 h asleep per night), and sleep inertia. Six patients reported onset age during adolescence. Patients were asked to practice good sleep hygiene and to complete a sleep diary during the week before PSG recording to avoid sleep deprivation and large variations in sleep onset and offset. Six patients had mean sleep latency <8 min on the MSLT, of whom one had one SOREMP and none had two or more SOREMPs. All patients underwent a prolonged continuous PSG recording showing a total sleep time >11/24 h for eight patients. Sleep efficiency on the PSG was >90%, with a respiratory event index (apneas + hypopneas) <10/h, periodic leg movement index during sleep <10/h, microarousal index <10/h, and normal percentages of slow wave and REM sleep. CSF lumbar puncture was performed in all cases and all had normal hypocretin-1 level (>200 pg/ml).

As controls, 19 healthy subjects [16 males and 3 females, median age 36 years (range 17–78)] participated in the study. All controls had normal clinical neurological examination, normal and regular sleep, no sleepiness complaint, no hallucinations, no sleep paralysis, normal body mass index, and were free of any treatment that may interfere with sleep, motor, or psychological functions.

None of the participants had any psychiatric disorder (especially depression) based on the DSM-V criteria (19). Patients were not taking psychostimulant medications or any other medications known to influence sleep ≥2 weeks prior to sleep recording and ^18^FDG-PET scanning.

All participants gave their written informed consent to participate in this study approved by the Montpellier University Hospital’s ethics committee.

### PET Procedure and Data Acquisition

All subjects (patients and controls) received *via* intravenous line an injection of 2.5 MBq/kg of FDG, between 10:30 a.m. and 12:30 a.m. Before the injection, subjects were comfortably seated in a quiet, isolated room and asked to keep eyes closed to avoid noxious, auditory, or visual stimuli, for durations of 5–10 min. Up to PET scan acquisition, patients and controls were asked to remain fully awake, to not move or talk, under the supervision of both a neurologist and a technician during the whole process. None of the subjects slept before the injection and during the PET scan acquisition as assessed by questioning the subject after the procedure. The vigilance state of patients and controls was also supervised clinically and by video by both a neurologist (Elisa Evangelista and Yves Dauvilliers) and a technician during the whole process. In addition, we controlled for the absence of emotional triggering factors during the entire process to avoid cataplexy-related episodes.

Positron emission tomography images were acquired 30 min after injection, during 15 min with a PET scanner Siemens mCT20 Flow. The volume was reconstructed using an iterative reconstruction (TrueX + TOF ultraHD PET with 8 iterations and 21 subsets, Gaussian 2 and zoom 2), following the manufacturer’s operating instructions. The resulting images contained 109 contiguous slices with a plane separation of 2 mm.

### Statistical Analyses

Categorical variables for the sample are presented as percentages, and quantitative variables as medians with range. Most distributions were skewed according to Shapiro–Wilks test. Skewed distributions between groups were compared using the Kruskal–Wallis test (multiple-group comparisons). Chi-square or Fisher’s tests were used to compare categorical variables between groups. When comparisons among the three groups were statistically significant, two-by-two comparisons were conducted with a multiple comparisons correction using the Bonferroni method.

Metabolic data were analyzed using SPM8 software (Wellcome Department of Cognitive Neurology, Institute of Neurology, London, UK) implemented in MATLAB (Mathworks, Sherborn, MA, USA). Images were spatially normalized into the ICBM standard space using 2nd degree B-spline interpolations, and smoothed using a 16-mm full-width half-maximum isotropic Gaussian kernel.

Areas of significant relative (instead of absolute) change between the groups (i.e., Narcolepsy, IH, and Controls) were estimated according to the general linear model using linear contrasts (14). Global metabolism adjustment was performed using proportional scaling. Main contrasts estimated the main effect of pathology [controls vs. narcolepsy; controls vs. IH] to identify the brain regions where glucose metabolism was decreased (or increased) in pathological populations as compared to the controls cohort. In a second step, the condition effects were assessed within the pathological populations (narcolepsy vs. IH) to identify brain areas where glucose metabolism was higher (or lower) as a function of the disease type. Given the extended age range in our populations (18–78 years), age was entered as a confounding covariate in all analyses. The resulting set of voxel values for each contrast constituted a map of the *T* statistics [SPM{T}], thresholded at *p*^unc^ ≤ 0.001 (*T* ≥ 3.35; uncorrected for multiple comparisons) or *p*^corr^ < 0.05 (corrected for multiple comparisons) in contrast between patient groups (narcolepsy or hypersomnia) and controls. Direct comparisons between pathological conditions were thresholded at exploratory level *p*^unc^ ≤ 0.05 (*T* ≥ 1.71; uncorrected). In all contrasts, minimal spatial extent for reported brain areas is 50 contiguous significant voxels. Previously used (20) liberal statistical thresholds (*p*^unc^ < 0.05) for the direct comparison between pathological conditions are justified by reduced sample size and variability in each subpopulation.

## Results

### Population

Demographic and sleep characteristics of patients with NT1, IH, and controls are summarized in Table 1 with statistics. Between-group gender differences were found: patients with narcolepsy and healthy controls were more likely to be men, and IH to be women. No significant between-group differences were found for age at time of study, age at onset of EDS, ESS, and body mass index. The mean sleep latency and the number of SOREMPs on the MSLT differed between patient groups as expected.

**Table 1:** Demographic, clinical, and sleep characteristics of patients with narcolepsy type 1 (NT1), idiopathic hypersomnia (IH), and healthy controls.

	NT1 *N* = 16		IH *N* = 9		Controls *N* = 19		*p*-Value
	*N*	%	*n*	%	*n*	%		
Sex							
Male	12	75.00	2	22.22	16	84.21	0.003
Female	4	25.00	7	77.78	3	15.79	
Age, in years^a^	30.00 (18.00–78.00)		27.00 (20.00–60.00)		36.00 (18.00–66.00)		0.84
BMI, in kg/m^2a^	23.80 (16.80–36.00)		24.32 (20.90–31.62)				0.40
Age at onset of EDS, in years^a^	17.50 (6.00–48.00)		17.00 (14.00–46.00)				0.70
Epworth sleepiness scale^a^	18.50 (13.00–22.00)		18.00 (11.00–24.00)				0.66
Hypnagogic hallucinations							
No	6	37.50	5	55.56			0.43
Yes	10	62.50	4	44.44			
Sleep paralysis							
No	10	62.50	8	88.89			0.16
Yes	6	37.50	1	11.11			
Multiple sleep latency test, in mn^a^	4.50 (0.40–9.80)		10.00 (1.40–12.60)				0.02
Number of SOREMPS^a^	4.00 (2.00–5.00)		0.00 (0.00–1.00)				<0.0001

*^a^Continuous variables were expressed in median (minimum value–maximum value)*.

### Cerebral Glucose Metabolism in Patients with NT1 and IH vs. Healthy Controls

**Figure 1:**
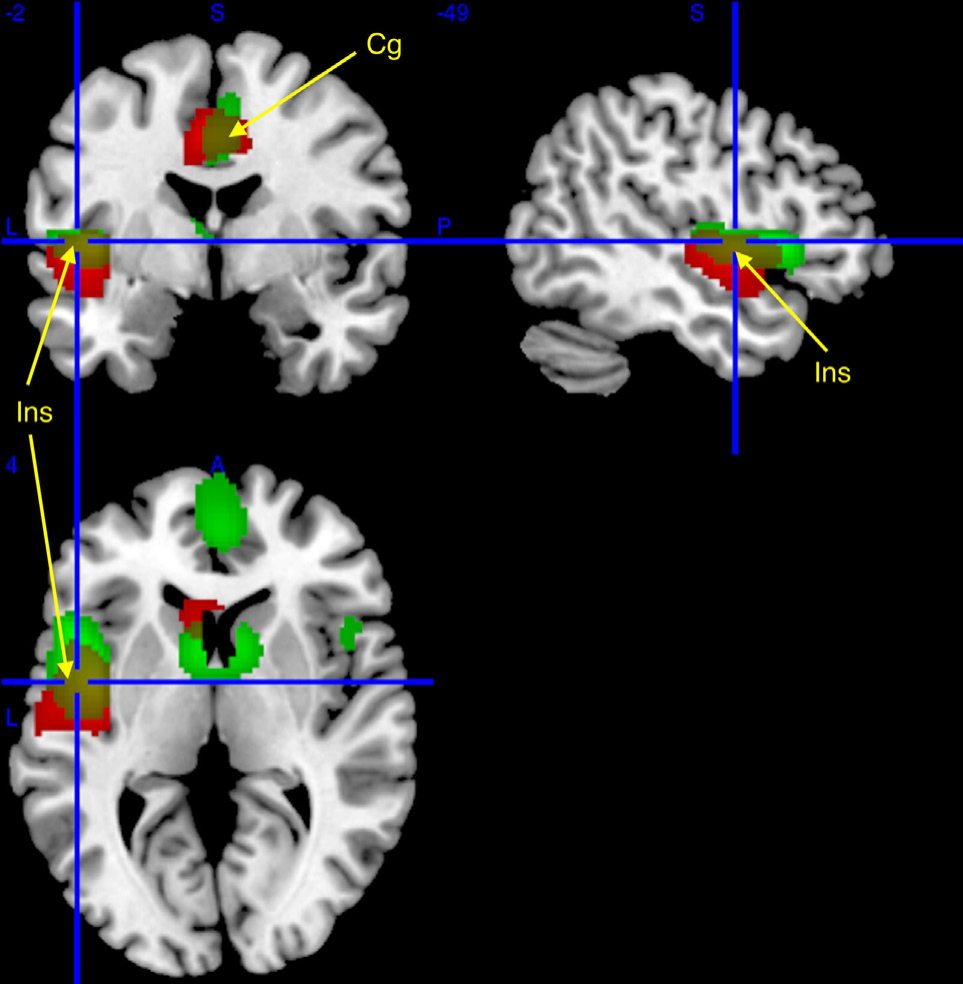
Regional cerebral increases in patients with narcolepsy type 1 (red) and idiopathic hypersomnia (IH) (green), as compared to healthy controls, superimposed on a template T1-weighted magnetic resonance image (MRI). Activations are displayed at *p*^unc^ < 0.001 uncorrected threshold, for clusters >50 voxels. Yellow clusters are area of similar hypermetabolism [insula (Ins) and middle cingulate (Cg); see arrows] in narcolepsy and IH as compared to controls.

In patients with narcolepsy as compared to healthy controls, regional glucose metabolism in the fully awake condition increased in the left rolandic operculum (*p*^corr^ < 0.05), and in the bilateral middle cingulate and fusiform regions, insula, and right temporal lobes (*p*^unc^ < 0.001; see Table 2 and Figure 1). No hypometabolism was found in patients with narcolepsy as compared to controls.

**Table 2:** Statistical parametric mapping results of brain regions showing relative metabolism changes in patients with narcolepsy type 1 (A) and idiopathic hypersomnia (IH) (B), as compared to healthy controls.

	Hemisphere	Coordinates			*Z*-score
	(L)eft/(R)ight	*x*	*y*	*z*	
**(A) Narcolepsy vs. healthy controls**					
**Hypermetabolism**					
Rolandic operculum	L	−44	−2	2	4.39***
Insula lobe	L	−42	8	−14	3.61
Middle cingulate	R	10	12	32	4.04
Middle cingulate	L	−4	2	40	3.98
Temporal pole	R	42	18	−22	3.65
Fusiform	R	40	−44	−16	3.62
Fusiform	L	−32	−46	−12	3.55
**Hypometabolism**					
No suprathreshold voxel					
**(B) IH vs. healthy controls**					
**Hypermetabolism**					
Insula lobe	L	−44	0	4	4.51***
Insula lobe	L	−44	8	2	4.50***
Insula lobe	R	46	20	−2	3.44
Caudate nucleus	R	6	12	−2	4.27**
Middle cingulate	–	−2	8	38	4.25**
Anterior cingulate	–	0	48	16	4.16
Anterior cingulate	R	6	38	28	4.02
**Hypometabolism**					
No suprathreshold voxel					

Coordinates *x, y, z* refer to the standard Talairach and Tournoux (21) stereotactic space. Activation peaks’ significance is reported at the voxel-level, thresholded at *p*^unc^ ≤ 0.001, excepted ***p^corr^ < 0.05 and **trend *p*^corr^ < 0.06. Cluster extent is >50 voxels for all reported locations. Only the most representative voxels in each anatomical structure are displayed.

In patients with IH as compared to healthy controls, brain regional metabolism in the fully awake condition increased in the insula and in the right caudate nucleus (*p*^corr^ < 0.05), and in the middle and anterior cingulate cortices (*p*^unc^ < 0.001; Table 2; Figure 1). Also in this population, no hypometabolism was found as compared to controls.

### Cerebral Glucose Metabolism in Patients with NT1 vs. IH

**Figure 2:**
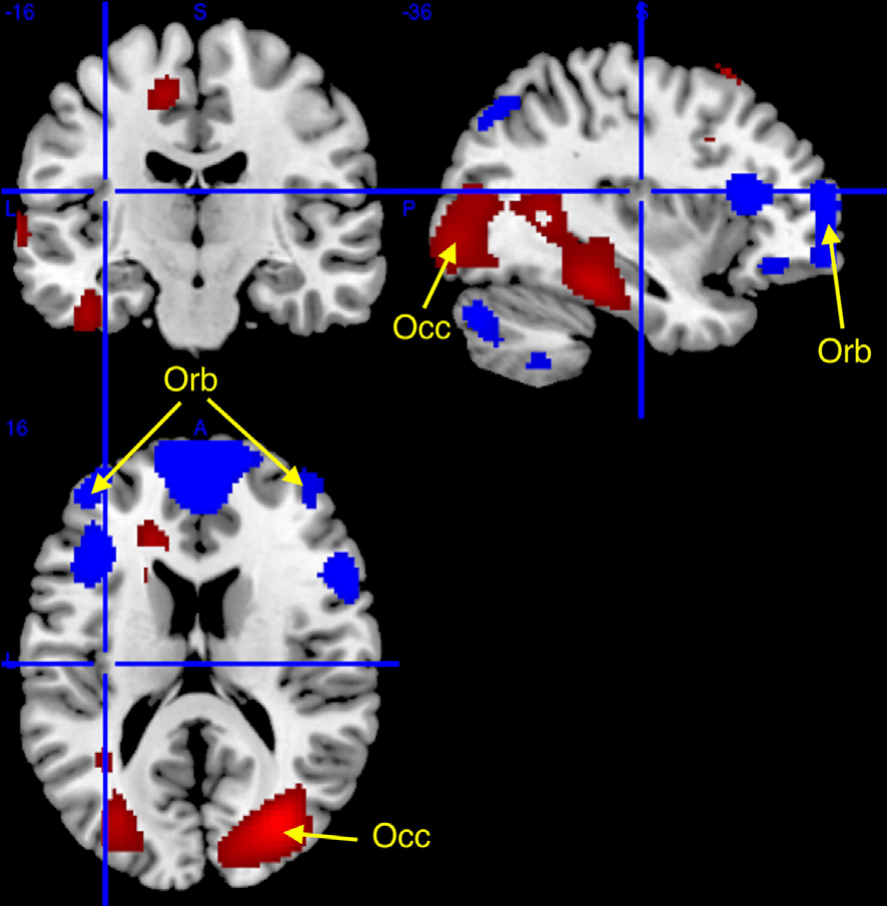
Higher (red) and lower (blue) regional cerebral glucose consumption in narcolepsy type 1 as compared to idiopathic hypersomnia, superimposed on a template T1-weighted magnetic resonance image. Higher metabolism (at *p*^unc^ < 0.001, cluster >50 voxels; see arrows) was found in narcolepsy in the superior occipital cortex (Occ) and lower metabolism was found bilaterally in the middle orbital gyrus (Orb) and in the supplementary motor area (not displayed). Other activations are displayed for the sake of completeness at *p*^unc^ < 0.05 uncorrected threshold, for clusters > 50 voxels.

Comparisons between patient groups were non-significant. However, slight between-groups differences were evidenced at conventional uncorrected statistical threshold (*p*^unc^ < 0.001), with higher glucose metabolism in narcolepsy than in IH in the right superior occipital gyrus, and conversely higher metabolism in IH than in narcolepsy in the bilateral middle orbital cortex and left supplementary motor area (Table 3; Figure 2).

**Table 3:** Statistical parametric mapping results of brain regions showing relative metabolism changes in patients with narcolepsy type 1 as compared to patients with idiopathic hypersomnia (IH).

	Hemisphere	Coordinates			*Z*-score
	(L)eft/(R)ight	*x*	*y*	*z*	
**(A) Narcolepsy vs. IH**					
Superior occipital	R	30	−76	16	0.001**
Middle occipital	L	−32	−76	16	0.010
Fusiform	R	34	−52	−10	0.014
Fusiform	L	−36	−34	−18	0.007
Supramarginal	R	36	−44	30	0.024
Middle temporal	L	−46	−52	6	0.002
Caudate nucleus (body)	L	−20	20	16	0.037
Middle frontal	L	−32	16	66	0.005
Medial frontal	L	−12	−20	54	0.009
Superior frontal	L	−18	32	62	0.015
**(B) IH vs narcolepsy**					
Middle orbital	R	48	56	−6	3.39**
Middle orbital	L	−48	50	−2	3.26**
Supplementary motor area	L	−6	10	72	3.22**
Inf. frontal (pars triang.)	L	−52	18	4	2.42
Middle frontal	R	34	6	56	1.88
Middle cingulate cortex	–	−4	−30	36	2.34
Inferior parietal	L	−54	−28	46	2.33
Inferior parietal	R	54	−62	52	2.48
Angular gyrus	R	56	−50	32	2.25
Angular gyrus	L	−38	−68	48	1.90
Precentral	L	−58	12	38	2.21
Precuneus	R	−6	−76	54	2.07
Postcentral	L	−16	−48	70	1.92
Caudate nucleus (head)	R	10	10	2	2.06
Caudate nucleus (head)	L	−6	8	2	2.01
Cerebellum (tonsil)	R	50	−60	−34	2.35
Cerebellum (pyramis)	R	48	−72	−34	2.35
Cerebellar (culmen)	–	0	−52	−24	2.22
Cerebellum (tuber)	L	−34	−78	−30	2.04
Cerebellum (tonsil)	L	−44	−54	−46	1.99

Coordinates *x, y, z* refer to the standard Talairach and Tournoux (21) stereotactic space. Activation peaks’ significance is reported at the voxel-level, thresholded at *p*^unc^ ≤ 0.05 (**p^unc^ < 0.001), uncorrected, with a cluster extent >50 voxels. Only the most representative voxels in each anatomical structure are displayed.

Further analyses conducted at a more lenient and exploratory uncorrected statistical threshold (*p*^unc^ < 0.05) indicated further differences between populations. In narcolepsy as compared to IH, metabolism was higher in the middle occipital and temporal, fusiform, medial, middle and superior frontal regions, and the body of the caudate nucleus (Table 3; Figure 2). Conversely, metabolism was higher in IH than narcolepsy in a set of brain areas encompassing the middle orbital cortex bilaterally, the supplementary motor area (SMA), the left pre- and post-central areas, the right precuneus, the inferior frontal and parietal regions, the angular gyrus, the cerebellum, and the head of the caudate nuclei, bilaterally (Table 3; Figure 2).

## Discussion

We report here a case-control ^18^FDG-PET scan study in drug-free patients with NT1, IH, and healthy controls. Our results evidence hypermetabolism in the two pathological conditions as compared to healthy controls, which involves similar regions namely the anterior and middle cingulate and the insula that are constitutive of the salience cortical network (22, 23). No hypometabolism was found in patients as compared to controls. Additionally, we compared patient groups with narcolepsy and IH and found no significant results. At sub-statistical, we found higher metabolism in the superior occipital gyrus in narcolepsy, and higher metabolism in the bilateral middle orbital cortex and left SMA in IH.

Functional MRI is the most commonly used method to identify resting-state brain networks. Alternatively, methods such as the FDG-PET may be useful to map resting brain functions while avoiding the temporospatial fluctuations inherent to the fMRI technique (24) and offering the injection and fixation of the radioisotope occurring outside of the scanner. Based on discordant PET-scan narcolepsy reports (2, 3, 6), we hypothesized that a large part of brain regional metabolism differences between patients with severe hypersomnia and healthy controls may actually reflect disturbances in the sleepiness-related neurocircuitry, that is, an alteration in the basic arousal system rather than a specific feature of the underlying disorder itself. Comparing well-characterized drug-free patients with NT1 and healthy controls, we showed in patients a significant hypermetabolism in a specific but widespread cortical network including the bilateral middle cingulate and fusiform regions, the rolandic operculum, and the temporal and insula lobes. These results confirm our preliminary findings in a limited sample of drug-free and treated patients with NT1 (2). A recent report also found that changes in brain metabolic activity in narcoleptic patients were positively correlated with results from the sleepiness scales and neurocognitive performance tests (6). We found a similarly increased metabolism in patients with IH (as compared to controls) in the middle and anterior cingulate, bilateral insula regions, and in the caudate nucleus. These results constitute to our knowledge the first neuroimaging evidence for similarly altered regional cerebral metabolism during wakefulness in two distinct central hypersomnolence disorders, and the first neuroimaging study conducted in patients with IH.

The topography of increased metabolism identified in both narcolepsy and IH is closer to the salience cortical network than to the default-mode and the executive-mode networks. Indeed, we especially found hypermetabolism in the anterior cingulate cortex and insula, which are key regions in the salience network (7, 22). The finding of an involvement of the salience network in the context of the present investigation is in good agreement with its known functional role (23). The salience processing requires the integration of sensory, visceral, autonomic and attention systems throughout the brain, and initiates appropriate responses to key stimuli as a function of top–down attention and cognitive control processes (23, 24). The salience network is also though to subtend the maintenance of tonic alertness, with correlations between its activity and upper alpha band oscillations (25). Another study found a predominance of sympathetic-associated regions in salience-processing networks, whereas parasympathetic regions predominated in the default mode network (26). Recent evidence suggest that alterations within the salience network contribute to deficits in social, affective, attention, and cognitive control processing in neurological and neurodevelopmental disorders (24). Such alterations in central disorders of hypersomnolence may contribute to a better understanding of the associated psychological symptoms (27).

In resting-state fMRI studies, methods and instructions given to subjects may vary widely (e.g., eyes open, closed, gaze fixation) eventually resulting in functional connectivity differences (28). The investigation of brain functional imaging in drug-free patients with central hypersomnolence disorders requires a specific instruction to “resist sleeping” to avoid monoamine neurotransmission and synaptic activity changes secondary to daytime sleepiness *per se*. All participants in the present study were scanned at rest but with similar instructions to not fall asleep during the PET procedure. The vigilance status during the scanning process may influence and raise some problems since subjects, especially drug-free patients affected with central hypersomnia, may present reduced attention and alertness leading to sleep onset. The increased metabolism identified in central hypersomnolence disorders close to the salience neural network reinforced its major role in the maintenance of the arousal level and the related sympathetic nervous system. This relative increased regional metabolism may thus be interpreted in this context as reflecting the patients’ motivation and cognitive effort to maintain alertness, as already reported in obstructive sleep apnea (29) and in Kleine–Levin syndromes (30). Sleep-deprivation studies in normal controls reported that poor performance elicit an attentional recovery that may manifest as greater activation in main areas such as the prefrontal and cingulate regions (31). Indeed, emerging evidence suggests that alterations in the coupling of brain networks (i.e., default-mode and salience networks) might be critical in cognitive alterations reported after sleep deprivation (31, 32). The salience network may thus play an important role in initiating network switching as a function of sleep propensity underlying the “wake state instability” model (33). A complex study using simultaneous fMRI-EEG recently investigated resting state brain function in adolescents with narcolepsy and showed altered brain dynamics that mainly relate to the default mode network (34).

Regional metabolic differences between the two patient groups were markedly restricted. In narcolepsy, there was increased metabolism in the middle occipital lobe (and to a lower extent in temporal, fusiform, medial, and superior frontal regions and the body of the caudate nucleus). In IH, increased metabolism was found in the middle orbital cortex and SMA (and to a lower extent in the pre- and post-central areas and the precuneus). The functional significance of these relative changes remains highly speculative, especially for areas where differences were found at lenient, unconventional statistical thresholds, and should be investigated in further studies. Notwithstanding, these findings may suggest an increased metabolism in several regions of the executive-control network in IH (35, 36). In contrast to patients with narcolepsy with often irresistible sleep as well as short and refreshing naps, patients with IH often complain of the non-refreshing quality of napping, sleep drunkenness, and persistent drowsiness (37). The metabolic differences between the two disorders within the executive-control network may be the signature of the abnormal neural system leading to the persistent sleep inertia, typical of IH. Our between-group finding differences may also indicate different modes of counteraction of hypersomnolence. Further works are required to validate the PET scan signature of sleep inertia, together with behavioral signs and EEG findings (38–40).

The present study has some strengths and limitations. Only typical and homogeneous drug-free patients with NT1 and IH were included and compared to healthy controls with similar instructions given during the scanning acquisition. While being fully awake during the whole process as controlled clinically and by video, unfortunately, the vigilance state was not monitored through EEG recording during the PET procedure. Our design procedure cannot directly affirm the absence of short change in alertness and even short sleep events during the whole scanning process. Further simultaneous EEG-monitored PET studies based on large samples are needed to confirm our preliminary results on IH, and to compare patients with central hypersomnia with healthy subjects in rested wakefulness and following a night of total sleep deprivation.

To conclude, we have evidenced a significant hypermetabolism in NT1 and IH in the wake resting state in regions constitutive of the salience cortical network, in comparison to healthy controls. The fluctuations of the brain’s intrinsic activity in both conditions may reflect the vigilance status during the scanning process rather than the underlying deficient system. The current use of metabolic neuroimaging cannot yet contribute significantly to perform central hypersomnia diagnosis but may provide useful information regarding the resting organization of the brain during drowsiness and sleep inertia.

## Ethics Statement

All participants gave their written informed consent to participate in this study approved by the Montpellier University Hospital’s ethics committee.

## Author Contributions

YD participated in the conception, design of the study and recruitment of subjects, analyzed and interpreted the data, and wrote the first draft of the manuscript. PP analyzed and interpreted the data, and revised the manuscript. DV performed the experiment and revised the manuscript. EE and LB participated in the recruitment of subjects for the study and revised the manuscript.

## Conflict of Interest Statement

We declare no conflicts of interest related to this article. YD received funds for seminars, board engagements, and travel to conferences by UCB Pharma, Jazz, Theranexus, Flamel, and Bioprojet. PP, DV, EE, and LB have no disclosure.
